# Sunitinib treatment in patients with advanced renal cell cancer: the Brazilian National Cancer Institute (INCA) experience

**DOI:** 10.1590/S1677-5538.IBJU.2015.0226

**Published:** 2016

**Authors:** Rafael Corrêa Coelho, Tomás Reinert, Franz Campos, Fábio Affonso Peixoto, Carlos Augusto de Andrade, Thalita Castro, Daniel Herchenhorn

**Affiliations:** 1 Departamento de Oncologia Clínica. Instituto Nacional do Câncer José de Alencar Gomes da Silva (INCA), RJ, Brasil; 2 Departamento de Urologia - Instituto Nacional do Câncer José de Alencar Gomes da Silva (INCA), RJ, Brasil; 3 Departamento de Estatística - Centro de Pesquisa Clínica (CPQ) - Instituto Nacional do Câncer José de Alencar Gomes da Silva (INCA), RJ, Brasil

**Keywords:** Neoplasm Metastasis, Kidney Neoplasms, sunitinib [Supplementary Concept], Retrospective Studies

## Abstract

**Purpose:**

The aim of this study was to assess the impact of sunitinib treatment in a non-screened group of patients with metastatic renal cell cancer (mRCC) treated by the Brazilian Unified Health System (SUS) at a single reference institution.

**Material and Methods:**

Retrospective cohort study, which evaluated patients with mRCC who received sunitinib between May 2010 and December 2013.

**Results:**

Fifty-eight patients were eligible. Most patients were male 41 (71%), with a median age of 58 years. Nephrectomy was performed in 41 (71%) patients with a median interval of 16 months between the surgery and initiation of sunitinib. The most prevalent histological subtype was clear cell carcinoma, present in 52 (91.2%) patients. In 50 patients (86%), sunitinib was the first line of systemic treatment. The main adverse effects were fatigue (57%), hypothyroidism (43%), mucositis (33%) and diarrhea (29%). Grade 3 and 4 adverse effects were infrequent: fatigue (12%), hypertension (12%), thrombocytopenia (7%), neutropenia (5%) and hand-foot syndrome (5%). Forty percent of patients achieved a partial response and 35% stable disease, with a disease control rate of 75%. Median progression free survival was 7.6 months and median overall survival was 14.1 months.

**Conclusion:**

Sunitinib treatment was active in the majority of patients, especially those with low and intermediate risk by MSKCC score, with manageable toxicity. Survival rates were inferior in this non-screened population with mRCC treated in the SUS.

## INTRODUCTION

Renal cell cancer (RCC) represents 2-3% of all cancers. Patients are diagnosed with locally advanced (stage III) or metastatic (stage IV) disease in approximately 33%, and 40% of those treated with curative intent surgery experience recurrence (1). Without treatment, the prognosis for metastatic renal cell cancer (mRCC) patients is restricted, with a median survival ranging from 6 to 12 months and a survival rate in two years between 10 and 20% (2). Immunotherapeutic agents, such as interleukin-2 (IL-2) and interferon-alpha (IFNα), were historically the only therapeutic options available for mRCC, despite the low response rates and a limited impact on overall survival (OS) (3–6).

The better understanding of the biological mechanisms related to carcinogenesis and intracellular signaling pathways enabled the creation of new treatment strategies for mRCC, with the introduction of targeted therapies. Sunitinib was identified as an inhibitor of platelet-derived growth factor receptors (PDGFRα and PDGFRβ), vascular endothelial growth factor receptors (VEGFR1, VEGFR2 and VEGFR3), stem cell factor receptor (KIT), Fms-like tyrosine kinase-3 (FLT3), colony stimulating factor 1 receptor (CSF-1R), and the glial cell line-derived neurotrophic factor receptor (RET). The inhibition of these tyrosine kinase (TK) receptors affects cellular signal transduction, thus influencing the processes involved in tumor growth, systemic dissemination and angiogenesis (7, 8).

The biological rationale for the use of VEGF pathway blocking agents for RCC is explained by the fact that the RCC is a highly vascularized tumor with high levels of VEGF and VEGFR expression. Furthermore, RCC is associated with mutations and/or defects in Von Hippel-Lindau (VHL) gene function and hypoxia-inducible genes, resulting in increased production of hypoxia-inducible factor (HIF), VEGF and PDGF (8, 9).

Motzer et al. randomized 750 treatment-naive RCC patients to receive sunitinib or IFNα in a prospective, phase III trial. Sunitinib treatment was associated with a higher objective response rate (47% versus 12%, p<0.001), leading to a median progression-free survival (PFS) of 11 months in the sunitinib arm, compared to 5 months in IFNα arm (p<0.001). The overall survival (OS) was 26.4 months in sunitinib arm and 21.8 months in IFNα arm (HR 0.82 p=0.051) (10, 11). Cella et al. demonstrated gain in quality of life for sunitinib, when compared to IFNα, and that the patients achieving a better quality of life had a longer progression-free survival, while the presence of hepatic metastases and a higher number of risk factors, as per Memorial Sloan Cattering Cancer Center (MSKCC) risk score, at the start of study were correlated with a shorter progression-free survival (12–14). Patients in the sunitinib arm experienced the following events as most common grade 3-4 toxicities: systemic hypertension occurred in 12% of the patients, fatigue in 11%, diarrhea in 9% and hand-foot syndrome in 9%.

The purpose of this study was to assess the impact of sunitinib treatment in terms of OS, PFS, and toxicity in a non-screened group of patients with mRCC treated by the Brazilian Unified Health System (SUS) at a single reference institution, while assessing the reproducibility of the clinical trial results in patients from routine clinical practice.

## MATERIALS AND METHODS

Between May 2010 and December 2013, 65 consecutive patients provided informed consent for the treatment of metastatic renal cell carcinoma with sunitinib at our institution (Clinical Oncology Service – Brazilian National Cancer Institute (INCA) – Rio de Janeiro, Brazil) and had their medical records reviewed. This study was approved by the Ethics in Human Research Committee of INCA and conducted in accordance with the Declaration of Helsinki and Good Clinical Practice guidelines.

We performed a retrospective cohort study. Clinical data including demographics, Eastern Cooperative Oncology Group (ECOG) performance status (PS), Memorial Sloan Kettering Cancer Center (MSKCC) risk classification for mRCC, stage, histology, previous therapies, and the toxicity related with sunitinib therapy were collected.

Response to treatment was assessed using clinical and, especially, radiological criteria as follows: complete response (CR), partial response (PR), progressive disease (PD), and stable disease (SD). The radiological evaluation was based on the Response Evaluation Criteria in Solid Tumors, version 1.1 (15), with a frequency determined by the assistant physician. The medium interval between the radiological evaluations was 3 months.

In our institution we standardized the evaluation of toxicities by the National Cancer Institute Common Toxicity Criteria, version 3.0, every month (16).

Exclusion criteria included patients without sufficient records to fill the questionnaire and another primary neoplasm except non-melanoma skin cancer.

Patients who were treated with other first line therapy than sunitinib and subsequently received the referred drug were not evaluated for PFS and OS, as well patients with histology of non-clear cell carcinoma. Toxicities were evaluated in all patients independent of histology and line of treatment.

Overall survival was estimated from the time of the first palliative treatment day until death or, for living patients, the last available follow-up, and PFS was measured from the date of the sunitinib treatment beginning to either first progression or death or the date of last contact for patients who were alive and progression-free, in both cases using the Kaplan-Meier method. Survival curves were compared by Log-Rank test. Association between response rate and MSKCC risk classification was analyzed by Fisher's Exact test. All analyses were performed with the SPSS software, version 18.0.

## RESULTS

Sixty-five patients were evaluated for this analysis; however, only 58 were eligible: 7 patients were excluded due to insufficient data in their records for the application of the research protocol.

Most of eligible patients were male – 41 (71%), with a median age of 58 years (18-80) and the majority underwent previous nephrectomy – 41 (71%), with a median interval between surgery and treatment beginning of 16 months (1-180). The most prevalent histological subtype was clear cell carcinoma, present in 52 (91.2%) patients.

The other histological subtypes found were: chromophobe cell in 1 patient (1.8%) and papillary cell in 3 patients (5.3%). One patient had a mixed tumor with papillary and clear-cell renal carcinoma characteristics (1.8%). Two patients from this cohort had partial responses and three had stable disease with sunitinib treatment.

The most frequent metastatic sites were lung, present in 42 patients (72%), followed by bone in 26 patients (45%), lymph nodes in 21 patients (36%) and liver in 9 patients (15%). Table-1 summarizes patients and tumor characteristics.

**Table 1:** Patients characteristics.

		n	%
**Age, years**			
	Median	58.0	
	Range	18−80	
**ECOG PS**			
	0-1	37	64
	2	12	20
**Histology**			
	Clear cell	52	91
	Non-clear cell	6	9
**Previous nephrectomy**		41	71
**Metastatic sites**			
	Lung	42	72
	Bone	26	45
	Lymph nodes	21	36
	Liver	9	15
	Adrenal glands	5	9
	Pancreas	4	7
	Locoregional	4	7
	Pleura	3	5
	Brain	2	3
**Previous systemic therapy** **a**,*			
	No previous systemic therapy	50	86
	Antiangiogenic b	3	5
	Cytokine	7	12
	mTOR inhibitor c	2	3
**Risk group according MSKCC criteria** **d**			
	Low risk	19	33
	Intermediate risk	23	38
	High risk	16	28
**Total**		**58 patients**	

a Includes 3 (7%) patients that participated in clinical trials

b Include sorafenib and bevacizumab.

c Include everolimus and temsirolimus.

d The MSKCC modified risk factors are PS ECOG ≥2, anemia, hypercalcemia, increased lactate dehydrogenase and time between nephrectomy and treatment shorter than 12 months [5]. MSKCC: Memorial Sloan-Kettering Cancer Center.

The standard dose of sunitinib used was 50mg orally once daily for four weeks, with two-weeks intervals (scheme 4:2). There was a change in the dose or regimen due to side effects in 24 patients (41%): 19 patients (33%) used 50mg in 2:1 scheme, three patients (5%) used the every-other-day scheme and 2 patients (3%) used 25mg a day on a continuous basis.

Nine patients began the treatment with 50mg in 2:1 scheme due to their borderline PS.

All the patients were assessed for toxicity. The most common adverse effects found were fatigue (57%), hypothyroidism (43%), diarrhea (29%), skin changes such as yellowing of the skin and rash (29%), mucositis (33%), hand-foot syndrome (HFS) (29%), hypertension (24%) and nausea (24%). Grade 3 and 4 adverse effects were infrequent: fatigue (12%), hypertension (12%), thrombocytopenia (7%), neutropenia (5%) and HFS (5%). Only one patient of those who experienced severe neutropenia had fever associated, with the need for hospital admission. Table-2 describes the treatment toxicities.

**Table 2:** Prevalence and grade of adverse effects (%)*

Adverse effect	Grade			
	All	1	2	3
Fatigue	57	21	24	12
Diarrhea	29	9	17	3
Skin changes	29	11	16	2
Mucositis	33	17	16	NA
Hand-foot syndrome	29	19	5	5
Hypertension	24	3	9	12
Nausea	24	15	9	NA
Anemia	19	3.5	12	3.5
Thrombocytopenia	15	3	5	7
Epistaxis	9	3.5	3.5	2
Neutropenia	10	2	3	5
Edema	5	2	3	NA
Neuropathy	5	3	2	NA
Change in Taste	5	3	2	NA
Constipation	5	2	3	NA
Bleeding	5	2	3	NA

* Considering valid information

NA, Not applicable

Rare adverse effects, possibly related to the use of the medication, that were not described in the tables below were: one acute coronary syndrome (G4), one transaminases elevation (G2), one nephropathy (G1), two deep vein thrombosis with pulmonary thromboembolism (G3, G3) and one anastomosis dehiscence (G3).

With the objective of avoiding confounding factors in the main analysis, only patients with renal clear cell carcinoma who were exposed to sunitinib as the first line of palliative treatment were evaluated for response, PFS and OS. A total of 45 (76%) patients were included in this analysis. Eighteen (40%) had partial response (PR), sixteen (35.6%) stable disease (SD) and 11 (24.4%) progressive disease (PD). Thus, approximately, 75% of the patients obtained clinical benefit with sunitinib treatment. The response rates were also assessed according to MSKCC risk groups, outlined in Table-3.

**Table 3:** Response rates assessed according to MSKCC risk groups.

			Response			
			Total	PR	SD	PD
MSKCC criteria	Favorable risk	Number of patients	**16**	10	5	1
		(%)	**35.6%**	55.6%	31.3%	9.1%
	Intermediate risk	Number of patients	**16**	6	7	3
		(%)	**35.6%**	33.3%	43.8%	27.3%
	High risk	Number of patients	**13**	2	4	7
		(%)	**28.9%**	11.1%	25.0%	63.6%
Total		**Number of patients**	**45**	**18**	**16**	**11**
		**(%)**	**100%**	**40%**	**35.6%**	**24.4%**

p-value = 0.003 by Fisher's Exact test; **PR** = partial response; **SD** = Stable disease; **PD** = Progressive disease **MSKCC** = Memorial sloan cattering cancer center (MSKCC)

**Figure 1:**
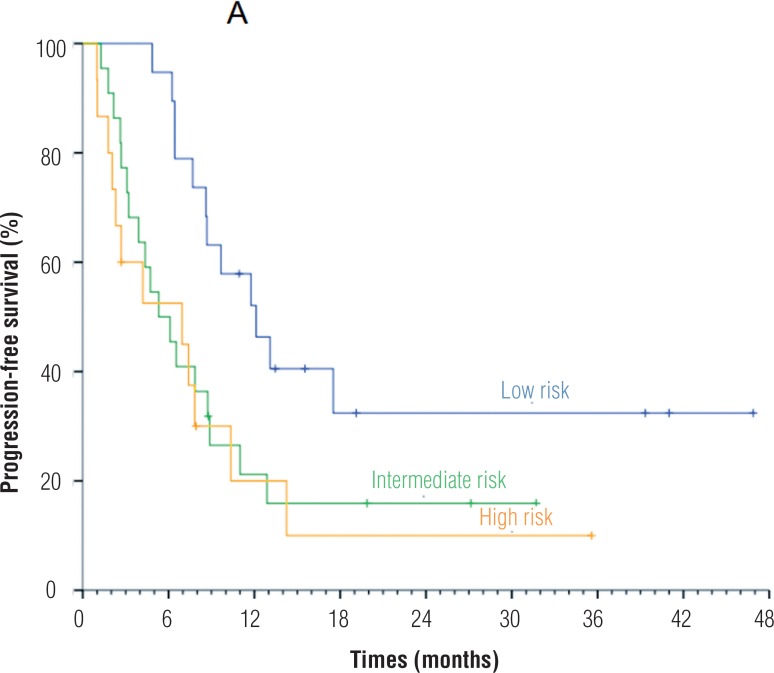
Progression free survival according to MSKCC risk groups. p-value = 0.029 by Log Rank test

We performed the PFS estimative in the 45 patients eligible to analysis and according to MSKCC risk groups of these patients. The global PFS was 7.69 months. When applying MSKCC criteria, patients with favorable, intermediate and high risk had a PFS of 8.9 months, 5.1 months and 2.6 months, respectively, with statistically significant difference between favorable and high risk groups (Figure-1). The median OS was 14.1 months, without statistically significant variation among risk groups.

Among the 45 eligible patients for PFS and OS analysis, we found six with PFS greater than 20 months. Three (50%) had MSKCC low risk, two (33.3%) intermediate risk and one (16.7%) high risk. The most common metastatic sites in this subset of patients were: lymph nodes (50%), lungs (50%), bones (33%) and locoregional (16.7%).

In eight patients (14%), sunitinib was the second or third line of systemic treatment. Among those who underwent previous treatment, four patients had partial response, one stable disease and two progressive disease with sunitinib treatment. Seven patients received interferon alpha, two received the combination of everolimus and bevacizumab in a clinical trial, one patient was treated with IFN and sorafenib and one patient sorafenib and one patient with temsirolimus.

Only 9 (16%) patients underwent a subsequent line after sunitinib treatment. m-TOR inhibitors were the most frequently used agents: 2 patients received temsirolimus and 5 patients everolimus. Furthermore, one patient received sorafenib and another pazopanib. The low rate of treatments after sunitinib progression occurred because at Brazilian public health facilities like INCA, second or third line treatments for mRCC are not routinely available.

## DISCUSSION

We reported a retrospective analysis of the use of sunitinib in advanced renal cell cancer patients treated at INCA's clinical oncology outpatient department. All patients were treated at the public health setting under the Brazilian Unified Health System (SUS).

The retrospective nature of this study raises the possibility of bias once some clinical details were not identified on the medical chart reviews. It is important to emphasize that our data express INCA's reality and, probably, the reality of other Brazilian and Latin America health institutions. So, the data in this article is of a great value and can help other institutions to organize their budget.

Firstly, the population is representative of the daily clinical practice in the public oncology services in Brazil. The population has characteristics that are different from the patients represented in prospective clinical trials. A higher proportion of factors associated with a poorer prognosis is seen, such as MSKCC high risk group, higher proportion of elderly patients and patients with performance status equals to or higher than 2 and/or who did not were submitted to previous nephrectomy.

The safety and tolerability was similar to that described in the medical literature (11, 12, 17-19). Most adverse events were mild and did not prevent the continuity of sunitinib treatment. Approximately 41% of the patients had their therapeutic regimens changed due to intolerance or by decision of the physician in charge, probably related to the performance status and comorbidities. Most of them were treated with regimen adjustment (50mg with a 2:1 interval), keeping the dose intensity. The rates of dose change are similar to literature data (17–19).

Sunitinib was a well-tolerated drug for most patients with 0-2 performance status; however, in patients with PS≥3, it had more harmful effects and severe toxicities, including death.

The median PFS and OS were lower than prospective data, probably because the population was non-screened, with a higher frequency of poor prognosis factors and lower frequency of second-line treatments. Nevertheless, a high response and clinical benefit rate (>75% of patients) was found. There is a risk that such data is overestimated by the methodology used in this study. Not surprisingly, patients with low risk, as per MSKCC score, had a higher rate of partial response and stable disease, as well a trend towards longer overall survival and a statistically significant better progression-free survival compared with high risk patients. We suggest that, in our population of patients with mRCC, clear cell histology and MSKCC low to intermediate risk, sunitinib is an active agent for first line therapy. Nevertheless, in MSKCC high risk patients, because response to sunitinib was poor, alternative treatments like temsirolimus need also to be considered (20).

Table-4 compares the results of this study with the results of studies performed in other countries and the controlled prospective study performed by Motzer et al. (11), which led to the approval of the drug.

**Table 4:** Comparison among studies evaluating sunitinib treatment in metastatic renal cell cancer.

Site	N	Prognostic factors	G3-4 Toxicities	Response Rate	PFS (m)	OS (m)
Coelho et al.	58	28.9% MSKCC high risk, 36% PS≥2, 27% ≥ 65 years old, 29 % without nephrectomy	12% fatigue, 12% hypertension, 7% thrombocytopenia, 5% neutropenia, 5% hand-foot syndrome.	Assessed in 45 patients: 40% partial response, 35.6% stable disease	7.6	14.1
Motzer et al. (11, 12)	375	10% without nephrectomy, 6% MSKCC high risk, PS 0-1, median age of 62 years	12% hypertension, 11% fatigue, 9% hand-foot syndrome and 9% diarrhea.	47%	11	26.4
Gore et al. (16)	4564	11% without nephrectomy, 9% MSKCC high risk, 14% PS≥ 2, 32% age ≥ 65 years	8% fatigue, 8% thrombocytopenia, 6% neutropenia, 6% asthenia, 6 % HFS, 5 % diarrhea	1% complete response, 16% partial response, 59% stable disease	10.9	18.4
Ansari et al. (17)	56	14 % without nephrectomy, 18% PS≥2, median age of 61 years, 50% received interferon alpha as previous treatment	21 % mucositis, 14% leucopenia, 13% neutropenia, 9% thrombocytopenia, 7% increased creatinine, 5% diarrhea, 5% hypertension, 5% HFS	Assessed in 49 patients: 41% partial response, 37% stable disease	12.2 no difference between patients receiving and not receiving immunotherapy	18.2 no difference between patients receiving and not receiving immunotherapy
Hong et al. (18)	76	Median age of 57.5 years, 10.5% PS =2, 10% MSKCC high risk, 4.3% without nephrectomy	38.2% thrombocytopenia, 10.5 % fatigue, 10.5% mucositis, 9.2 % HFS	27.6% objective response and 84.2% with controlled disease	7.2	22.8

Another factor that could be involved in the inferior survival is the absent information on second or third line therapies, as these therapies are not available for patients in the Brazilian Unified Health System (SUS), and most of the patients received only one line of treatment for metastatic disease. So, agents like everolimus, sorafenib and axitinib were not routinely prescribed (21–23). These agents showed activity in patients who failed to VEGF targeted therapies increasing progression free survival, but without impact in overall survival. Some reasons that could explain why OS benefit was not achieved in the studies evaluating the drugs described above are: study design and PFS as primary endpoint instead of OS, crossover between study groups and sequential treatments. Escudier et al. showed overall survival benefit of 3.5 months with sorafenib when post-crossover placebo survival data were censored, reinforcing our hypothesis (22).

## CONCLUSIONS

Sunitinib treatment was active in the majority of patients, especially those with low and intermediate risk by MSKCC score, with manageable toxicity. Furthermore, patients categorized as low-risk exhibited a trend towards higher response rate, longer progression-free survival and overall survival. Overall and progression-free survival for our patient's cohort were inferior when compared to phase 3 trials probably because the present study evaluated a non-screened population with mRCC treated at the Brazilian public health system.

## LIST OF ABBREVIATIONS

INCA: = The Brazilian National Cancer Institute
RCC: = Renal cell cancer
mRCC: = Metastatic renal cell cancer
IL-2: = Interleukin-2
IFNα: = Interferon-alpha
OS: = Overall survival
PFS: = Progression-free survival
PDGFRA AND PDGFRB: = Platelet-derived growth factor receptors
VEGFR1, VEGFR2 AND VEGFR3: = Vascular endothelial growth factor receptors
KIT: = Stem cell factor receptor
FLT3: = Fms-like tyrosine kinase-3
CSF-1R: = Colony stimulating factor 1 receptor
RET: = Glial cell line-derived neurotrophic factor receptor
TK: = Tyrosine kinase
MSKCC: = Memorial sloan cattering cancer center
SUS: = Brazilian unified health system
HFS: = Hand-foot syndrome
